# Tracking the impact of multiple sclerosis on employment status: the development of a questionnaire

**DOI:** 10.1055/s-0042-1755344

**Published:** 2022-11-09

**Authors:** Evangelina Valeria Cores, Judith Steinberg, Carolina Cuesta, María Celeste Curbelo, Mabel Alicia Osorio, Johana Julia Bauer, Daniel Gustavo Politis

**Affiliations:** 1 Consejo Nacional de Investigaciones Científicas y Técnicas, Buenos Aires, Argentina.; 2 Hospital Interzonal de Agudos “Eva Perón”, Laboratorio de Deterioro Cognitivo, Buenos Aires, Argentina.; 3 Universidad de Buenos Aires, Faculty of Psychology, Buenos Aires, Argentina.; 4 Hospital Británico, Demyelinating Diseases Area, Buenos Aires, Argentina.; 5 Esclerosis Múltiple Argentina, Buenos Aires, Argentina.

**Keywords:** Surveys and Questionnaires, Unemployment, Work, Multiple Sclerosis, Disability Evaluation, Pesquisas e Questionários, Desemprego, Trabalho, Esclerose Múltipla, Avaliação de Incapacidade

## Abstract

**Background** Multiple sclerosis (MS) has a negative effect on employment status.

**Objective** To present the preliminary results of a special questionnaire designed to collect employment information on patients with MS.

**Methods** The questionnaire on the impact of MS on employment status was completed by 63 patients. Fatigue, cognition, and depression were also evaluated, and 33 healthy participants were recruited as a control group.

**Results** Regarding the patients' employment status, we found rates of 31.7% of full-time employment, 28.6% of part-time employment, 7.9% of unemployment due to MS, 4.8% of housewives, 1.6% retirement due to age, 15.9% of retirement due to disability, 7.9% of medical leave due to MS, and 1.6% of medical leave for other reasons. The rate of unemployment among the patients was significantly higher compared with that of the control group. Out of 38 working patients, 31% had been absent from work for the previous 3 months due to MS, and 50% had to make changes in their work to remain employed. Out of the 19 unemployed patients, 78% said that walking difficulties were the cause of unemployment, while 52% thought cognitive impairment was the cause.

**Conclusions** The questionnaire provides a record of the employment status of patients with MS and describes the impact on work from their point of view.

## Resumo

**Antecedentes** A esclerose múltipla (EM) tem um efeito negativo na situação laboral.

**Objetivo** Apresentar os resultados preliminares de um questionário elaborado para coletar informações sobre a situação laboral de pacientes com EM.

**Métodos** O questionário sobre o impacto da EM na situação laboral foi preenchido por 63 pacientes. Fadiga, cognição e depressão também foram avaliadas, e 33 pacientes saudáveis foram incluídos como grupo controle.

**Resultados** Quanto à situação laboral dos pacientes, foram encontradas taxas de 31,7% de emprego em tempo integral, 28,6% de emprego em meio período, 7,9% de desemprego por EM, 4,8% de donas de casa, 1,6% aposentadoria por idade, 15,9% de aposentadoria por invalidez, 7,9% por licença médica por EM, e 1,6% de licença médica por outros motivos. A taxa de desemprego entre os pacientes foi significativamente mais alta em comparação com a do grupo controle. Dos 38 pacientes empregados, 31% tiveram que se afastar do trabalho nos 3 meses anteriores por causa da EM, e 50% tiveram que fazer mudanças em seu trabalho para permanecer empregado. Dos 19 pacientes desempregados, 78% mencionaram que as dificuldades de locomoção foram a causa do desemprego, enquanto 52% reportaram que a deterioração cognitiva foi a causa.

**Conclusões** Este questionário fornece um registro da situação laboral dos pacientes com EM e descreve o seu impacto no emprego do ponto de vista deles.

## INTRODUCTION

Multiple sclerosis (MS) is a chronic immunological disease in the central nervous system that mainly affects people of working age, and whose prevalence is estimated in 36 to 41 cases per 100,000 people in Buenos Aires, Argentina. 1 Having a job has benefits for self-esteem, and enables independent living and integration as a member of society. Multiple sclerosis has a negative impact on employment status, which, among other things, leads to difficulties in work, absenteeism, and job loss. 2 In 2016, the Multiple Sclerosis International Federation (MSIF) 3 released a report on MS and employment based on data from a global survey of more than 12,200 people affected by MS in 93 countries, with responses received mainly from Europe and North America. It indicated that 43% of unemployed participants had left work within 3 years of the diagnosis. This rate increased to 70% ten years after diagnosis.

In Argentina, Vanotti et al. 4 reported an unemployment rate of 30% in patients without cognitive impairment, and of 50% in patients with cognitive impairment, demonstrating a strong relationship between the socioeconomic status of patients and their employment status. Cores et al. 5 revealed an unemployment rate of 30% among patients attending public hospitals, and demonstrated the link between unemployment and the speed of the cognitive treatment. Through the Spanish adaptation of The Buffalo Vocational Monitoring Survey, 6 Vanotti et al. 7 found a rate of unemployment of 19.32%.

This type of information is widely used to develop therapeutic intervention strategies and health policies that can promote permanence in the workplace, as well as the opportunity to develop and advance in the job position, therefore reducing the negative impact of different aspects of the disease over the employment status of the patient. Appropriate instruments are needed to collect this data.

The purpose of the present work is to evaluate the employment status, work difficulties and their causes, and unemployment, as perceived by those diagnosed with MS, as well as other aspects such as the support or rejection they experience in their working environment, through a questionnaire designed for MS patients from Buenos Aires.

## METHODS

### Design

The present is a descriptive and inferential study with intentional and non-probabilistic sampling.

### Subjects

A total of 63 patients diagnosed with MS were recruited from specialized centers. The inclusion criteria for this group were: patients from 18 to 60 years of age, to have a MS diagnosis, and to have more than 6 years of schooling. The exclusion criteria were: presence of other diseases that may cause cognitive impairment, history of alcoholism or drug abuse, patients in relapse phase, use of corticosteroids in the last three months prior to the assessment, and presence of visual disturbances and/or serious motor disorders or psychiatric diseases, such as euphoria, major depressive disorder, bipolar disorder, pathological crying and/or laughter, and psychosis.

We also recruited 33 volunteer participants in the control group (CG). The inclusion criteria were: age from 18 to 60 years, and having more than 6 years of schooling. The exclusion criteria were: diagnosis of a condition that may cause cognitive impairment or hinder the perfomance of tests and the application of questionnaires, psychiatric illness, history of alcoholism or drug abuse, score lower than 27 points in the Mini-mental State Examination (MMSE), 9 10 and/or score higher than 15 points in the revised Beck Depression Inventory (BDI-II). 11

### Instruments

The following instruments were administered to patients with MS.

Based on the patients' responses when openly questioned about employment, we developed a questionnaire named “Tracking MS Impact on Work Status Questionnaire” (TMSWSQ), which contains questions about job status, causes of work difficulties, changes made because of the illness, negative symptoms, and more. The questionnaire was presented to five neurologists specialized in MS, who gave their opinion, on the basis of their own experiences, regarding the issues related to the emplyments status of MS patients.

The questionnaire contains a core question about whether the subject was currently employed or held a paid job in the previous week. Those who responded affirmatively had to answer questions about: the type of work they were doing; the number of daily working hours; their degree of physical and mental stress; the number of times they had been absent from work in the three previous months and the reasons; how often had they been late or had to leave work early and the reasons; if their duties at work had changed (so they had to perform easier or lighter tasks, or had to start working remotely, or had their hours reduced, or if they needed more hours to complete a task); the main symptoms that affected their work; and if they felt supported or rejected by their colleagues and superiors.

The questionnaire identifies the following work status: employee, housewife, student, retired due to age, retired due to disability, and unemployed. In all cases, except for employed persons, it was asked whether MS was related to the employment status of the patient. Those who had retired early due to MS and those who were unemployed but had previously worked were asked about the symptoms that they felt had led them to lose their job.

In addition, the cognitive functions were measured through an MS neuropsychological screening battery 12 composed of: the Memory Selective Test (MST), which measures the acquisition of verbal episodic memory (MST learning) and its recovery (MST deferred memory); the 7/24 Spatial Recall Test (7/24 SRT), to assess visuospatial memory, with scores related to learning tasks and deferred memory; the Paced Auditory Serial Addition Test (PASAT), in its 3-second (PASAT-3) and 2-second (PASAT-2) versions, according to the presentation of the interstimulus interval; the phonemic verbal fluency test; and the Symbol Digit Modalities Test (SDMT). According to Cáceres et al., 13 a patient who obtained a score less than the 5th percentile of the CG in at least 2 tests, was considered to have a cognitive impairment.

Physical disability was assessed using the Expanded Disability Status Scale (EDSS). 14 Depression was assessed using e thBDI-II; scores ≥ 15 correspond to depression. The Fatigue Severity Scale (FSS) 15 was implemented to measure fatigue as perceived by the patient, in which significant fatigue corresponds to a score ≥ 4.

The CG was submitted to the aforementioned neuropsychological screening battery, the BDI-II, and the MMSE. In addition, they had to select their employment status from the following options: full-time, part-time, casual, housewife, student, retiree due to age, and retiree due to disability or medical leave. These subjects did not answer the TMSWSQ.

### Procedures

Approvals from the Ethics Committe were obtained in accordance with the rules and regulations of Hospital Interzonal de Agudos “Eva Perón.”

The evaluations were performed individually in one hour and a half by a qualified neuropsychologist. Previously, a specialist neurologist diagnosed and assessed the patients using the EDSS.

### Statistical analysis

Statistics, such as means, standard deviations (SDs), and frequencies were used to describe the sample. The Student independent sample *t* -test was used to calculate the distribution of the quantitative variables and to perform a comparison between the groups, while the Chi-squared (χ ^2^ ) test was used to calculate the distribution of the qualitative variables and to perform a comparison between the groups. A significance level of 0.05 was adopted.

## RESULTS

Of the 63 patients assessed, 55 had relapses and remissions, 5 had secondary progressive course, 2 had primary progressive course, and 1 had primary progressive course with relapses. The patient group had a mean age of 44.4 (SD = 10.7) years, with a mean of 13.6 (SD = 4) years of schooling, and a mean EDSS score of 2.6 (SD = 2). The CG had a mean age of 45.3 (SD = 10) years, and a mean of 14.9 (SD = 2.8) years of schooling ( Table 1 ).

**Table 1:** Demographics, clinical data, and employment status of the study sample

Variable	MS patients: mean (standard deviation)	Control group: mean (standard deviation)
Age (years)	44.4 (10.7)	45.3 (10)
Schooling (years)	13.6 (4)	14.9 (2.8)
EDSS score	2.6 (2)	−
FSS score	4.4 (1.6)	−
BDI-II M (SD) score	14 (10.2)	−
Female gender	58.7%	69.7%
Employment status: unemployed + disability retirement	31%	3%

Abbreviations: BDI-II, Beck Depression Inventory-II; EDSS, Expanded Disability Status Scale; FSS, Fatigue Severity Scale; MS, multiple sclerosis.

In the patient group, no significant differences were found regarding age groups (t = 0.381; df = 94; *p* = 0.704) and years of schooling (t = 1.711, df = 94, *p* = 0.090. 58.7%). In the CG, 69.7% of the participants were female, with no significant differences in terms of gender distribution (χ ^2^ = 1.111; gl = 1; *p* = 0.203; Fisher exact statistic).

Regarding the employment status of the patients, we found that 31.7% were employed full time (7 hours or more a day), 28.6% had part-time jobs (less than 7 hours per day), 7.9% were unemployed due to MS, 4.8% were housewives, 1.6% were retired due to age, 15.9% had disability retirement due to MS, 7.9% were on medical leave because of MS, and 1.6% were on sick leave for another reason. There were no students in the sample. In the CG, 66.7% were employed full-time, 15.2%, part-time, 6.1% were housewives, 9.1% were retired due to age, and 3% were on medical leave.

To compare the employment status between the groups, the variables were reclassified into 4 subgroups: 1. full-time and part-time employment; 2. housewives; 3. retired due to age; 4. unemployed + disability retirement + medical leave due to MS; 5. medical leave due to other reasons. Significant differences between the groups were found by recoding the variables, with a larger number of subgroup 4 participants in the MS group (31%) compared with the CG (3%) (χ ^2^ = 13.162; gl = 4; *p* = 011).

### Employment

Of the 38 full-time and part-time employees, 31% had to be absent due to MS in the previous 3 months, either due to a symptom (referred by 6 patients), medical appointments (7 patients), or medical examinations (3 patients). A total of 28% of the patients had to come late or leave work due to MS in the previous 3 months, either because of a symptom (referred by 4 patients), medical appointments (9 patients) or medical examinations (6 patients). 50% had to promote changes in their work to remain employed, such as performing simpler tasks (reported by 7 patients), less strenuous tasks (9 patients), working from home (2 patients), reducing working hours (4 patients), taking more time to perform some tasks (7 patients), take frequent breaks (9 patients), change the work schedule (3 patients), or implement some other type of change (2 patients).

Impacts of MS on work were reported by 73% of the patients group. The main aspects perceived as causes were difficulties in walking (reported by 11 patients), difficulties regarding mobility of the hands (5 patients), urinary incontinence (5 patients), pain (5 patients), fatigue (16 patients), cognitive impairment (13 patients), the fact that they had to attend medical examinations and consultations (8 patients), and emotional disorders (3 patients).

When asked if they thought they could be promoted or improve their work in any way without MS, 15 patients answered yes or maybe. Among those who had a superior partner or work partner, 6 patients responded that they felt rejected because of their MS, while 23 patients indicated that they felt their support.

### Causes of unemployment

When analyzing the causes perceived by patients regarding their employment status, 78% of the 19 patients in subgroup 4 mentioned that difficulties in walking, 47% reported hand mobility difficulties, 47% reported urinary incontinence, 57% reported fatigue, 47% reported pain, and 10%, emotional disorders.

Cognitive impairment was one of the reasons reported by 52% of patients who were unable to work due to MS. Among these patients, 9 reported difficulties due to memory loss, 4 said that there were problems with performing two tasks at the same time, 9 showed focused attention deficit, 5 had difficulty in language expression, 5 had disorders in comprehension, and 8, organizational difficulties.

The relationship between the presence of objective cognitive impairment and the perception of cognitive difficulties as a cause of unemployment was not significant (χ ^2^ = 0.031; gl = 1; *p* = 0.430 (Fisher exact statistic). Of those who reported that cognitive impairment was the cause of unemployment, 7 had objective cognitive deficits, and among those who did not consider it a cause of unemployment, 5 had objective cognitive deficits.

### Unemployment and clinical-demographic variables

Regarding employment status, no significant association was found regarding subgroups 1 ( *n* = 38) and 4 ( *n* = 19), and the presence of cognitive impairment, (χ ^2^ = 1.062; gl = 1; *p* = 0.151) (Fisher exact statistic) or depression, (χ ^2^ = 0.171; gl = 1; *p* = 0.453) (Fisher exact statistics). A significant association between employment status and presence of fatigue was recorded (χ ^2^ = 0.627; gl = 1; *p* = 0.215) (Fisher's exact statistic).

Out of the 19 subjects in subgroup 4, 13 had cognitive impairment, 8 had depression (score > 15 points on the BDI-II), and 11 showed fatigue (score > 4 points on the FSS).

Unemployed patients ( *n* = 19) were older, had lower levels of schooling, and higher scores on the FSS and EDSS. No significant differences were found regarding the BDI-II scores between the groups. These data are presented in Table 2 .

**Table 2:** Comparison of demographic and clinical variables of employed and unemployed patients

Variables	Patients with multiple sclerosis		Comparison statistic		
	Employed: mean (standard deviation)	Unemployed: mean (standard deviation)	t	gl	*p*
Age (years)	40.8 (9.8)	51 (8.6)	3.882	56	0.000
Schooling (years)	14.5 (3.5)	12.3(4.8)	-2.013	56	0.049
EDSS score	2.1 (1.6)	3.8 (2.2)	2.695	56	0.012
FSS score	4.4 (1.6)	3.5 (1.4)	2.117	56	0.038
BDI-II score	14 (10.2)	18.1 (12)	1.326	56	0.190

Abbreviations: BDI-II, Beck Depression Inventory-II; EDSS, Expanded Disability Status Scale; FSS, Fatigue Severity Scale.

In conclusion, the TMSWSQ provides a sufficiently detailed record of the employment status of people with MS and how work is affected by MS, as well as the causes of unemployment and the symptoms or aspects of the disease that affect the working conditions as perceived by patients. An understanding of these issues provides guidelines for interventions aimed at reducing unemployment and the impact of MS on work.

The TMSWSQ is the first questionnaire developed in the region based on the experience of professionals specialized in MS which examines conflicts that patients have experienced during medical consultations. When asked openly about their opinion of the questionnaire after they took it, the patients stated orally that their difficulties were comprehensive. In a final question open to all other work difficulties not included in the questionnaire, with a written answer, no patient added any point other than those contained in the items of the questionnaire, which confirms, at least on a preliminary basis, that it covers the issue.

According to the results obtained in the present study, the reported unemployment rate (30%) is similar to the one reported by Vanotti et al., 4 who showed that 10 patients with cognitive impairment and 17 patients without it were unemployed or retired from a total of 82 patients diagnosed with MS, that is, 32% of the sample were unemployed or retired. In addition, they showed associations between employment status and depression, and fatigue and physical disability. In the present study, no significant associations were found between employment status and depression, although significant associations were found with fatigue and physical disability.

On the other hand, among the most significant results of the present study is the description of how people who work feel that MS affects their work, with symptoms of fatigue, cognitive impairment, and walking difficulties considered the most influential by the patients. Not all patients who identified cognitive impairment as one of the causes of their employment status objectively had deficits in the neuropsychological tests performed, possibly because cognitive complaints do not correlate with objective performance in patients with MS. 16

As a result of the data herein reported, patients had to make changes to keep their jobs. These are the most reported changes: performance of less demanding physical tasks, taking more time to perform certain tasks, and taking more frequent breaks. Among the unemployed, early retirees and patients on medical leave due to MS, walking difficulties and fatigue were the symptoms most often perceived as causes behind their employment status. Despite the small number of housewives included in the sample, those who participated did not mention a relationship between MS and their employment status. Environmental changes such as the ability to take breaks, work from home, provide means of transportation and adequate infrastructure to transfer the patient from home to work, as well as mobility in the workplace, could help patients keep their jobs.

Another important fact uncovered by the present study is that a small portion of the patients felt rejected because of MS in their work environment. Nevertheless, those who had decided not to inform their colleagues or bosses of their disease did not answer this particular question. This could potentially overlap with the social impact of MS, and it underlines the need for further investigations on discrimination and employment.

In the present article, the data was collected from two specialized MS centers in Buenos Aires. However, it is important to continue to conduct epidemiological studies on this topic throughout the country, as the working conditions in the different regions are different, so the effects of MS may also vary from region to region. The interventions to be developed should be as specific as possible and take into account the needs of the patients.

In the future, psychometric studies will be required to generate quantitative variables to assess the degree of impact of MS on employment status.

Similarly, the use of the questionnaire herein presented in long-term studies may enable the creation of risk profiles for loss of employment and data on how and when the employment status of MS patients changes.
